# Optimization of SPME-Arrow-GC/MS Method for Determination of Free and Bound Volatile Organic Compounds from Grape Skins

**DOI:** 10.3390/molecules26237409

**Published:** 2021-12-06

**Authors:** Iva Šikuten, Petra Štambuk, Jasminka Karoglan Kontić, Edi Maletić, Ivana Tomaz, Darko Preiner

**Affiliations:** 1 Department of Viticulture and Enology, Faculty of Agriculture, University of Zagreb, 10 000 Zagreb, Croatia; pstambuk@agr.hr (P.Š.); jkkontic@agr.hr (J.K.K.); emaletic@agr.hr (E.M.); itomaz@agr.hr (I.T.); dpreiner@agr.hr (D.P.); 2 Centre of Excellence for Biodiversity and Molecular Plant Breeding, Faculty of Agriculture, University of Zagreb, 10 000 Zagreb, Croatia

**Keywords:** SPME-Arrow, grape skins, volatile organic compounds

## Abstract

(1) Background: Solid phase microextraction (SPME)-Arrow is a new extraction technology recently employed in the analysis of volatiles in food materials. Grape volatile organic compounds (VOC) have a crucial role in the winemaking industry due to their sensory characteristics of wine.; (2) Methods: Box–Behnken experimental design and response surface methodology were used to optimise SPME-Arrow conditions (extraction temperature, incubation time, exposure time, desorption time). Analyzed VOCs were free VOCs directly from grape skins and bound VOCs released from grape skins by acid hydrolysis.; (3) Results: The most significant factors were extraction temperature and exposure time for both free and bound VOCs. For both factors, an increase in their values positively affected the extraction efficiency for almost all classes of VOCs. For free VOCs, the optimum extraction conditions are: extraction temperature 60 °C, incubation time 20 min, exposure time 49 min, and desorption time 7 min, while for the bound VOCs are: extraction temperature 60 °C, incubation time 20 min, exposure time 60 min, desorption time 7 min.; (4) Conclusions: Application of the optimized method provides a powerful tool in the analysis of major classes of volatile organic compounds from grape skins, which can be applied to a large number of samples.

## 1. Introduction

Grapevine is one of the most important horticultural crops in the world, mainly used for wine production. Thus, the aroma and flavor of wine are one of the main characteristics that define the differences among the vast array of wines and wine styles produced throughout the world [1] and in essence the wine producers are selling a sensory experience to the consumers [2]. The aromatic profile of grapes is very complex and includes a large number of different volatile organic compounds (VOCs). Even though the overall volatile composition of most grape varieties is similar, the aroma largely derives from differences in the relative ratios of many VOCs [3]. The main groups of volatile compounds found in grapes are terpenoids (monoterpenes, sesquiterpenes), norisoprenoids (mainly C_13_-norisoprenoids), volatile phenols, alcohols, carbonyls, and methoxypyrazines.

VOCs in grapes are present in free and bound forms, which are usually bound to a sugar moiety and are ten times more abundant [4]. VOCs are distributed in both the flesh and the skin of the berry, though their content in the skin is much higher [5]. The glycosidically bound VOCs can be released by acid or enzymatic hydrolysis. The products from a chemical acid hydrolysis may reflect more closely the natural states of free aromatic compounds in grapes and wine because wine is produced under acidic conditions [4]. This was shown by Loscos, et al. [6], who compared enzymatic and acid hydrolysis. Even though the enzymatic hydrolysis showed higher efficiency, the levels of most VOCs found were poorly correlated with those found after alcoholic fermentation. On the other hand, the transformations taking place during fermentation include relevant chemical rearrangements in acid media that are better predicted by acid hydrolysis.

To analyze volatile compounds from grapes several extraction techniques are used: liquid–liquid extraction (LLE), solid phase extraction (SPE), solid phase microextraction (SPME), stir bar sorptive extraction (SBSE). These techniques are usually coupled with GC/MS instruments [7]. Among these techniques, the SPME has emerged as one of the preferred extraction methods for analysis of grape and wine VOCs [8,9,10,11,12,13,14,15,16] due to simplicity, accuracy, and reliability. In this technique fused silica fibre that is coated on the outside with the different stationary phase is a key element of extraction. Sample preparation is simple when this technique is fully automated [17]. There are several types of stationary phases such as polydimethylsiloxane (PDMS), polyacrylate (PA), divinyl-benzene (DVB), carboxen (CWR) and their combinations (CWR/PDMS, DVB/CWR/PDMS, PDMS/DVB) which are commercially available. Thickness and type of stationary phases determine fibre properties in terms of polarity and retention [18]. The PDMS is a non-polar phase which is preferred for the extraction of non-polar analytes. The PA is more polar than PDMS and it is preferred for the extraction of polar analytes. Fibres with the mixed coat increase retention capacity due to the mutually potentiating effect of the adsorption and distribution to the stationary phases [18]. There are some drawbacks associated with SPME fibre. Among them, the most pronounced are limited mechanical robustness and lack of physical durability of the fused silica [19], poor inter-device reproducibility, and small extraction phase volume [20]. Recently, a new technology called SPME-Arrow was introduced, which combines the advantages of SPME and SBSE techniques [21]. SPME-Arrow is coated with a larger amount of sorbent material than the traditional SPME fibre, allowing for more volatile compounds to be extracted and analyzed [22]. Furthermore, SPME-Arrow can also be fully automated [23].

Risticevic, et al. [19] described all parameters and steps which must be taken into account during the optimization of SPME method. The parameters are as follows: type of fibre coating, extraction mode, separation and detection, agitation method, analyte derivatization, sample volume or weight, pH, ionic strength, water content, organic solvent content, extraction temperature and time and desorption conditions. These parameters are strongly dependent upon the type of sample.

Recently SPME-Arrow has been employed in the analysis of volatile compounds in food materials. The analysis has been carried out on salmon, mushroom [24], fish samples [25,26], soy sauce [27], vinegar [17], milk [28] and distillates [23,29]. Regarding the wine volatiles, the published work is scarce and includes the work of Lisanti, et al. [30]. The work focuses on optimising SPME-Arrow conditions for the analysis of terpenoids, compounds that could contribute to the mint aromas of red wine bouquet. However, there are virtually no reports on SPME-Arrow analysis of volatile compounds from grape samples. Even though the above-mentioned work focuses on wine volatiles, the optimized method is not usable for grape samples. While grapes are solid matrices, wine is a complex liquid matrix that has been affected by numerous processes during vinification, such as maceration, alcoholic fermentation, or malolactic fermentation. All these processes alter the VOCs and thus do not represent the grape volatile profile. Thus, the aim of this work was to develop and validate SPME-Arrow sampling technique coupled with GC/MS instrument for the analysis of free and bound volatile organic compounds from grape skins.

## 2. Results and Discussion

### 2.1. Determination of Sample Weight

**Figure 1:**
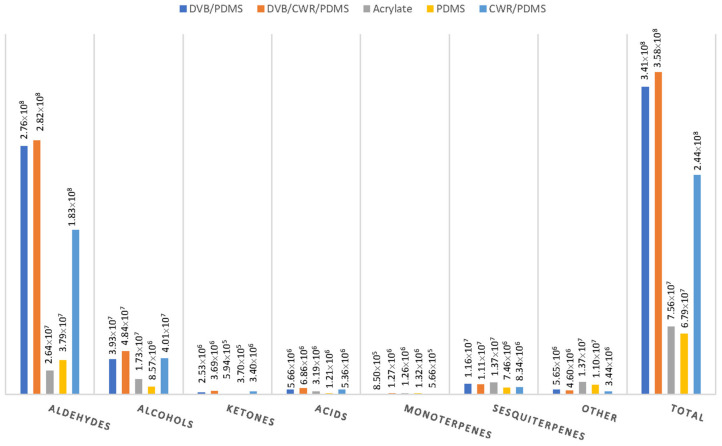
The effect of sample weight and mode of injection on GC/MS instruments on absolute peak areas.

Sample weight is one of the most important parameters which greatly influence extraction efficiency. It is well known that the amount of analytes extracted from the sample increases with the sample size. This parameter is more prominent for the compounds having high distribution coefficient between coating and the sample. In a case of the compounds with the small value of the distribution coefficient between coating and the sample, the amount of analyte extracted is almost independent upon sample weight [19]. For SPME fibre technique it is recommended to use splitless mode of injection [18]. SPME-Arrow has 6 to 20 times larger volume of the sorption phase in comparison with the conventional SPME, thus, to overcome potential overload of GC column sometimes split mode is used [17,22,25]. For the above-mentioned reason, before starting the optimization process, the appropriate sample weight and injection mode on GC/MS instruments had to be determined. For sample weight 100, 300, and 500 mg were chosen, while injection mode was tested in split and splitless (1:5) mode. The results obtained in these one-factor-at-a-time experiments are presented in Figure 1.

First all sample weights were tested in split mode, where higher sample weight resulted in higher peak areas. The statistically significant difference in peak areas is observed for all analyzed groups of compounds except acids. Based on this observation it can be assumed that all analyzed compounds except acids have high distribution coefficient between coating and the sample. As SPME-Arrow has greater adsorbing capacity in comparison with the SPME fibre, it was assumed that the great majority of adsorbed analytes are not directed to the GC column in split mode, thus, to test this hypothesis the splitless mode was chosen. Since the sample weight of 100 mg gave satisfactory results, meaning that the number of detected compounds did not change, it was tested in splitless mode. As can be seen on Figure 1, the splitless mode gave the highest peak areas for all analyzed classes of volatiles. Thus, the sample weight of 100 mg and splitless mode of injection on GC/MS instruments were used in further optimization processes.

### 2.2. Selection of the SPME-Arrow Fibre Coating

Five commercially available fibre coatings were tested for their extraction efficiency of VOCs from grape skins. The extraction efficiency of SPME depends greatly on the value of the distribution constant of analytes partitioned between the sample and the fibre coating material [11]. According to the literature PDMS is non-polar phase and it can be used for volatile, non-polar analytes, PA is polar phase and it can be applied for isolation of polar, semi-volatile analytes, CWR/PDMS is bipolar phase which is preferentially used for very volatile analytes, DVB-PDMS is also bipolar phase which can be applied for extraction of aromatic, semi-volatile analytes, and DVB/CWR/PDMS has polar and non-polar components and thus is wildey applied for analysis of volatile and semi-volatile analytes with wide range of polarity [31]. Thus, the selection of a suitable fibre coating is an important step in the extraction process.

**Figure 2:**
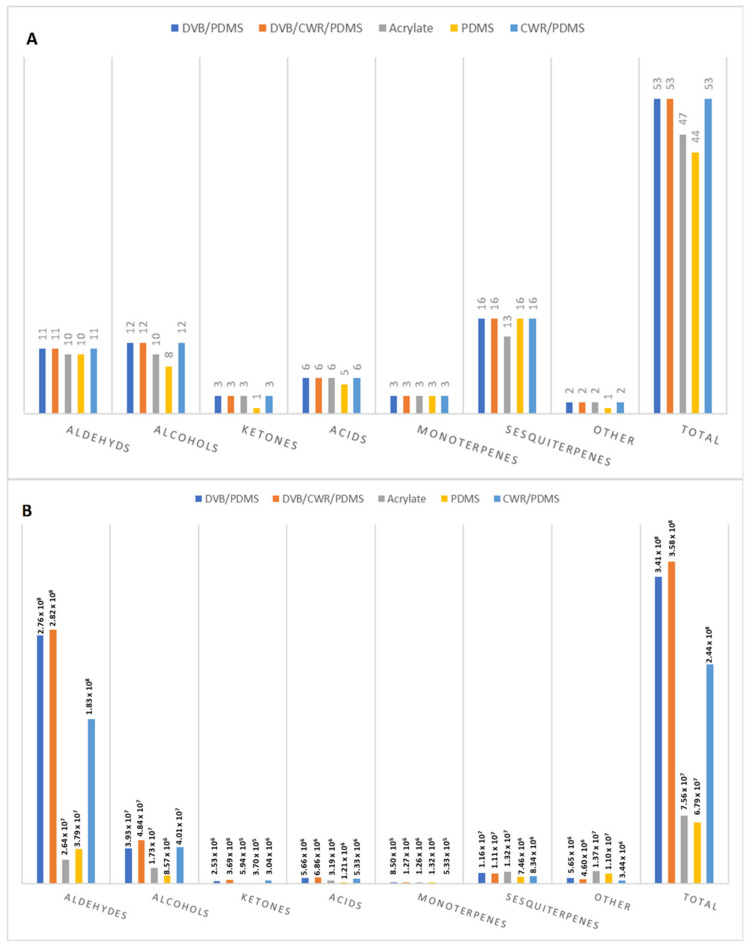
Number of identified volatile compounds (**A**) in skins of cv. Merlot, and the absolute peak areas (**B**) using five different SPME Arrow fibre coatings.

A total of 53 volatile compounds were extracted and identified using five commercially available fibres (Supplemental Table S1), belonging to the following groups: aldehydes (11 compounds), alcohols (12 compounds), ketones (3 compounds), acids (6 compounds), monoterpenes (3 compounds), sesquiterpenes (16 compounds), and other (2) compounds. In Figure 2A,B the number of compounds detected and the extraction efficiencies of SPME fibres expressed as GC absolute peak area are represented. A slightly smaller number of detected compounds was recorded for PDMS (44 compounds detected). The undetected compounds belong to aldehydes, alcohols, ketones, and acids and have Log Kow values in range from 0.79 to 2.73, which means that they are quite polar. The partition coefficient between octanol and water is a measure of compound polarity and it serves as a measure of the relationship between hydrophobicity and hydrophilicity (water solubility) of a compound. Measured values of Kow for organic chemicals have been found as low as 10^−1^ and as high as 10^7^, thus encompassing a range of ten orders of magnitude. In terms of Log Kow, this range is from −1 to 7. The higher the values, the corresponding compound is more hydrophobic e.g., less polar. In addition, these compounds are present in low content. These results are in agreement with the fact that PDMS is a non-polar phase. In a case of PA, 47 compounds were detected. The great majority of undetected compounds belong to sesquiterpenes which are non-polar compounds, as a result their affinity for PA as polar phase is low. The fibres differed more in the extraction efficiencies. The best results, with highest peak areas, gave DVB/CWR/PDMS fibre, followed by DVB/PDMS. These two fibres showed the highest peak areas for all the classes of VOCs, and the total peak area of 3.58 × 10^8^ for three-phased fibre and 3.41 × 10^8^ for DVB/PDMS. CWR/PDMS fibre showed relatively lower peak areas with a total peak area of 2.44 × 10^8^. The PA and PDMS fibres had the lowest peak areas for all the classes of VOCs. These results are in accordance with the properties of analytes presented in terms of Log Kow and vapour pressure (Supplemental Table S1). The vapour pressure is defined as the pressure exerted by a vapour in thermodynamic equilibrium with its condensed phases (solid or liquid) at a given temperature in a closed system. The equilibrium vapour pressure is an indication of a liquid’s evaporation rate. It relates to the tendency of particles to escape from the liquid (or a solid). A substance with a high vapor pressure at normal temperatures is often referred to as volatile. For the identified compounds vapour pressures are in the range from 0.025 up to 1280 Pa, which means that the sample is composed from semi-volatile and volatile compounds. Based on the results, the three-phased DVB/CWR/PDMS fibre was selected for further SPME-Arrow optimization.

### 2.3. Optimization of SPME-Arrow Conditions

The experimental variables included in BBD experimental design were chosen by taking into account that these variables could greatly influence the equilibrium of the volatile compounds in the headspace of the sample [19]. For instance, it is known that an increase in extraction temperature can release more volatiles to the headspace due to the effect on the vapour pressure and enhance the overall extraction procedure [28]. In addition, extraction time is an important parameter because headspace SPME is an equilibrium process of analytes between fibre and vapour phase [32].

In this study the evaluated experimental factors were temperature (40, 50, 60 °C), incubation time (10, 20, 30 min), exposure time (30, 45, 60 min), and desorption time (5, 7.5, 10 min). The BBD experimental design generated 27 experiments, including three central points (C). The experiments were randomly performed. To evaluate the significance of each of the studied factors, the area of the responses based on the sum of the peak areas was used. This is one of the most useful parameters for the optimization of the SPME conditions [29,33]. Fitting the data with various models showed that the content of aldehydes, alcohols, ketones, acids, monoterpenes and sesquiterpenes were best described with a quadratic polynomial model. For models, analyses of variance (ANOVA) parameters are presented in Table 1. The determination coefficients (R^2^) are in range from 0.9379 to 0.9919, while *p*-values are in range from <0.0001 to 0.0332 indicating that models are highly significant. Models also showed statistically insignificant lack of fit because all determined *p*-values are higher than 0.4500. Parameters of quadratic polynomial equations for all responses are also presented in Table 1.

**Figure 3:**
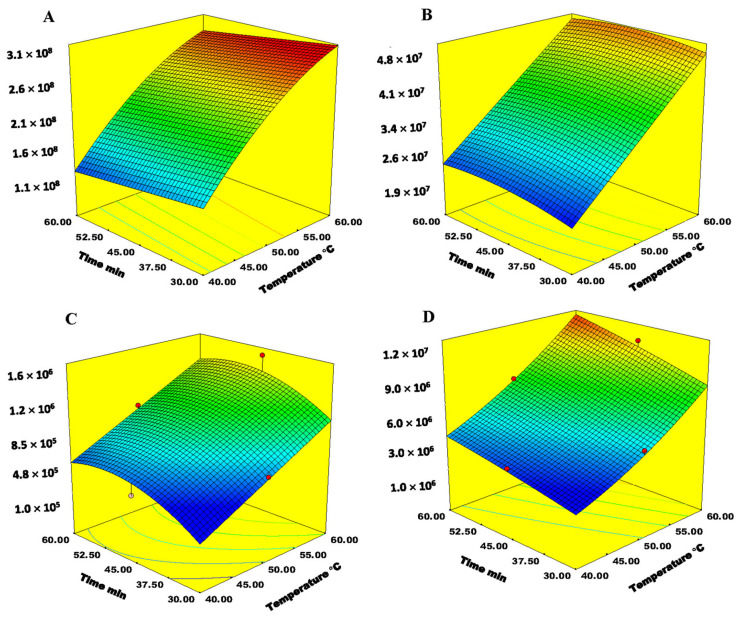
Response surface plot for interaction between extraction temperature and exposure time for free (**A**) aldehydes, (**B**) alcohols, (**C**) monoterpenes and (**D**) sesquiterpenes.

The results obtained in this study indicate that the most significant factors were extraction temperature (*p*-values for almost all group of analyzed VOCs are lower than 0.0001) and exposure time (*p*-values for all group of analyzed VOCs are in the range from 0.0002 up to 0.0412) (Table 1). An increase in extraction temperature positively influenced the extraction efficiency for aldehydes, acids, alcohols, monoterpenes, and sesquiterpenes. The rising temperature from 25 to 60 °C exponentially increased vapour pressure and thereby enhanced the volatilization. Based on the obtained results, the optimum extraction temperature is 60 °C. Similar results were obtained by Perestrelo, et al. [11], who also found that extraction at 60 °C gave the best extraction efficiency for VOCs from grape skins. Ketones were the only class of VOCs whose extraction efficiency was reduced by increasing temperature. The optimum temperature for ketones was 50 °C. However, the ketones were found in small amounts in grape berries, thus the extraction temperature of 60 °C was chosen as optimum temperature for extraction of VOCs from grape skins. The increase in exposure time positively affected extraction efficiency of individual classes of VOCs. The increase in exposure time positively affected extraction efficiency of aldehydes, acids and sesquiterpenes. An increase in exposure time up to 49 min positively influenced monoterpenes, ketones, and alcohols, while a further increase in exposure time ended up reducing the extraction efficiency. Thus, the optimum exposure time of 49 min was selected for all classes of VOCs. This indicated that the equilibration was achieved after 49 min and saturation of the stationary phase. After that, desorption can occur at a higher rate than adsorption. The incubation and desorption time did not have significant influence on the extraction efficiency. However, to visualize the effect of extraction temperature and exposure time, two closely connected factors, a surface model for all classes of VOCs was used (Figure 3). This interaction is statistically significant for aldehydes, alcohols, monoterpenes and sesquiterpenes. Aldehydes have vapour pressure determined at 25 °C in the range from 31 up to 1280 Pa (Supplemental Table S1.) with the average value of 349 Pa. Based on these values, these compounds are quite volatile even at room temperature, so the observation that the rising temperature up to 60 °C in the short exposure time gave maximal extraction efficiency is not a surprise. The analytical signals in a case of alcohols and monoterpenes tend to increase with an increase in extraction temperature and then slightly decrease with an increase in exposure time, with the maximal extraction efficiency achieved at around 45 min of exposure. Sesquiterpenes are the least volatile at room temperature. Their values of vapour pressures are in the range between 1 and 2 Pa. The observation that the temperature increases together with the prolonged exposure time have a positive effect on the extractability of sesquiterpenes could be explained by their low volatility. Efficiency of thermal desorption in a GC injector is strongly dependent upon analyte volatility, injector temperature, thickness of the arrow coating and exposure time. In theory, the optimal desorption temperature should be equal to the boiling point of the analyte with the highest boiling point [18]. In the case of analyzed compounds, the temperature of desorption should be 324 °C, but this is impossible because the recommended working temperature for the DVB/CWR/PDMS phase is between 220 and 300 °C. Long exposure of the arrow to temperatures close to the maximal recommended temperature have negative effect on the lifetime of the arrow. Taking into account the range of boiling points of analyzed compounds and recommended working temperatures for the DVB/CWR/PDMS phase, 250 °C was chosen as a compromise between optimal desorption and arrow protection from deterioration. Desorption time was not a significant parameter in the process of optimization.

The optimal conditions for SPME-Arrow extraction of free VOCs are presented in Table 2. These conditions were obtained by applying Derringer function or desirability function. The estimated and obtained values presented are similar, indicating good performance of the method developed for the extraction of free VOCs from grape skins. In addition, the calculated standard deviations based on the three measurements are low, indicating that the developed method is reproducible. Moreover, Merlot is an international variety and grown in many famous wine regions. Although it is considered a neutral grape variety considering its aromatic potential, Merlot contains volatile compounds that have important roles in grape and wine quality, such as aldehydes, alcohols or sesquiterpenes. Since most of the grape varieties are considered to be aromatically neutral and contain similar classes of volatiles to Merlot, the obtained and optimized conditions can be used in the analysis of other red grape varieties. Similarly, Merlot was used in the optimization process developed by Arcari, et al. [34], Welke, et al. [35]. 

### 2.4. Optimization of SPME-Arrow Conditions for Analysis of Bound VOCs

**Figure 4:**
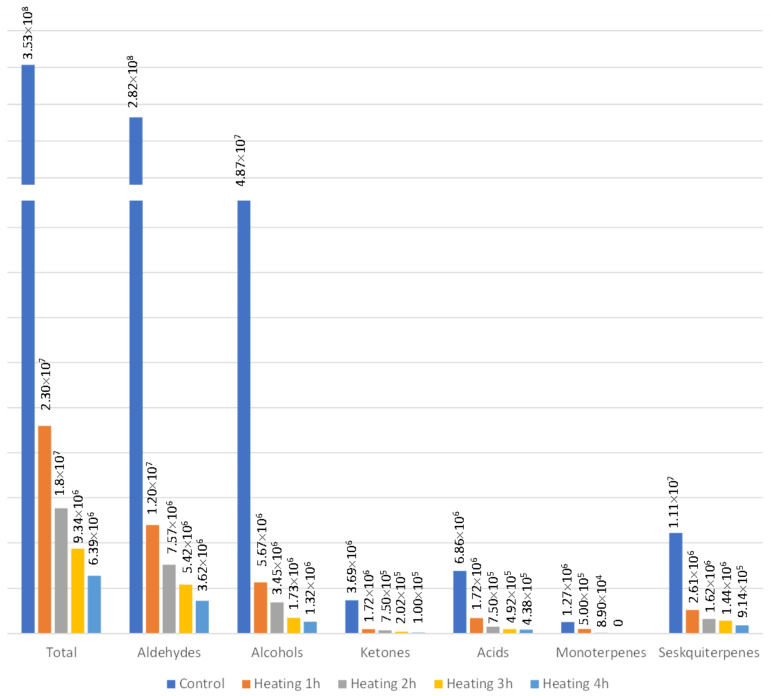
The effect of different heating times on different classes of free volatiles represented as absolute peak areas.

Before starting the optimization experiments, the heating of samples in order to remove free volatiles was carried out. The effect of different heating times on the content of different classes of free VOCs is presented on Figure 4. As expected, the prolonged heating time decreased the content of free VOCs. Thus, the heating time of 4 h was chosen as a suitable time for removing free VOCs from the sample. 

Acid hydrolysis is a complex process accompanied by several steps. In the first step, the solvation of dry grape skins occurs when skins are put in contact with the citric buffer. After solvation is complete, dissolution of bound forms of VOCs occurs. To enhance the process of hydrolysis, the reaction is conducted at elevated temperature (95 °C) in the nitrogen atmosphere to inhibit any oxidation reaction. Before performing the SPME-Arrow extraction procedure, it is necessary to increase the ionic strength of the sample solution. Aqueous solubility of many organic compounds decreases with the addition of the salt to the sample. The distribution coefficient between coating and the sample increases with the decreasing of aqueous solubility [19]. The effect of salting out leads to the more readily passage of the analyte from liquid sample to the headspace, and hence to the coating of SPME-Arrow. The weight of NaCl (2 g) was chosen based on the previously reported study [11].

Classes of bound volatile compounds identified in the Merlot grape skins were monoterpenes (21), C_13_-norisoprenoids (3), alcohols (33), acids (7), carbonyl compounds (18), and others (2). In total 84 volatile compounds were identified (Supplemental Table S2). The glycosidically bound monoterpenes are usually 3 to 10 times more abundant than free aglycones and the proportion of the glycosidic aroma substances changes among grape varieties [4,36]. Norisoprenoids are carotenoid-derived aroma compounds. During development carotenoids are degraded to produce glycosidically bound norisoprenoids [37]. Although the other classes of VOCs are mostly synthesized during alcoholic fermentation, they can also be found in bound forms in grape berries [38]. All detected compounds have a greater water solubility in comparison with the compounds detected as free direct from the solid matrix.

Similar to the optimization of SPME-Arrow conditions for analysis of free VOCs, the BBD experimental design was chosen. The experimental factors were again extraction temperature (40, 50, 60 °C), incubation time (10, 20, 30 min), and exposure time (30, 45, 60 min). Desorption time was not included, as it was shown that it did not affect the extraction efficiency, and was set to 7 min. The BBD experimental design generated 15 experiments including three central points. The experiments were again randomly performed. For the evaluation of the significance for all the studied factors, the area of the responses based on the sum of the peak areas was applied. Fitting the data with various models showed that contents of alcohols, carbonyls, acids, and monoterpenes were best represented by quadratic polynomial models. In Table 3 are presented parameters of analyses of variance (ANOVA) parameters for all studied models. The determination coefficients (R^2^) are in range from 0.8991 to 0.9931 while *p*-values are in range from <0.0001 to 0.0364, indicating that models are highly significant. Models also showed statistically insignificant lack of fit because all determined *p*-values are higher than 0.2500. All responses parameters of quadratic polynomial equations are depicted in Table 3.

**Figure 5:**
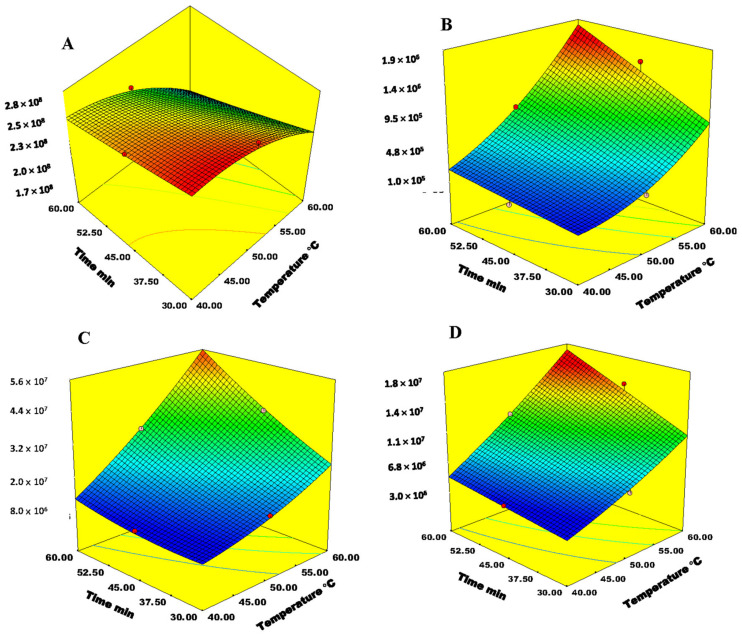
Response surface plot for interaction between extraction temperature and exposure time for bound (**A**) alcohols, (**B**) C13-norisporenoids, (**C**) monoterpenes and (**D**) carbonyls.

The results obtained showed that the most significant factors were extraction temperature and exposure time (Table 3). For both factors an increase in their values positively affected the extraction efficiency for almost all classes of VOCs. Thus, the extraction temperature of 60 °C and exposure time of 60 min were chosen. Incubation time again did not have significant influence on the extraction efficiency. The analytical signals tend to increase with a decrease in both extraction temperature and incubation time. In Figure 5 are presented 3D surface plots for the effect of extraction temperature and exposure time for all classes of VOCs.

This interaction is statistically significant for alcohols, C_13_-norisporenoids, acids and monoterpenes. Alcohols have vapor pressure with the average value of 93 Pa. Based on these values, these compounds are quite volatile even at room temperature, thus the observation that the rising temperature up to 60 °C in the short exposure time gave maximal extraction efficiency is not a surprise. The analytical signals in case of C_13_-norisoprenoids, acids and monoterpenes tend to increase with an increase in extraction temperature and with an increase in exposure time, with the maximal extraction efficiency achieved at around 60 min of exposure. Furthermore, the extraction temperature of 60 °C was chosen as an optimum, so a compromise had to be made. The incubation time did not show as a significant factor. The optimal conditions obtained by applying Derringer function or desirability function for SPME-Arrow extraction of bound VOCs are presented in Table 4. The estimated and experimentally obtained values presented are similar, indicating a good performance of the method developed for the extraction of bound VOCs from grape skins.

### 2.5. Method Validation

In the Supplemental Table S3 are presented the parameters of method validation. For all analyzed compounds good linearity could have been obtained. For the aldehydes LOQ was in the range from 2.21 up to 16.22 µg/kg. The highest LOQ was calculated for acids with an average value of 669.87 µg/kg. The RSD values obtained for intraday and interday precision did not exceed 15%. The method can be considered accurate because accuracy values for all analyzed compounds were within 15% of the nominal value.

## 3. Materials and Methods

### 3.1. Materials and Reagents

Grape samples of cultivar Merlot were obtained in 2020 from the vineyards located at the Experimental station ‘Jazbina’, Faculty of Agriculture, University of Zagreb, Zagreb, Croatia. The samples were collected at maturity state based on acid/sugar ratio. The basic chemical parameters were: total soluble solids 95° Oechsle, titratable acidity 6.62 g/L, pH 3.42. Ten grape clusters were randomly selected, weighing approximately 1.5 kg, from the vines taking into consideration the balance between shadow and sun exposure in the different vineyard locations. The batch of 300 berries was removed from the clusters using small scissors and were left with attached pedicles and frozen in liquid nitrogen. The berries weighed 384.60 g. The frozen samples were stored at −20 °C until further use. The grape skins were manually removed from frozen berries and freeze-dried. To obtain powder the skins were grinded using MiniG Mill (SPEX SamplePrep, Metuchen, NJ, USA), and were stored at −20 °C.

### 3.2. SPME-Arrow and GC/MS Analysis

The SPME-Arrow extraction was carried out using RSH Triplus autosampler (Thermo Fisher Scientific Inc., Brookfield, MO, USA). The appropriate sample weight was placed in 20 mL headspace screw-top vials sealed with PTFE/silicone septum containing caps.

Separation and detection of the samples was carried out by TRACE^TM^ 1300 Gas Chromatographer coupled to ISQ 7000 TriPlus quadrupole mass spectrometer (Thermo Fisher Scientific Inc., Bartlesville, OK, USA) equipped with TG-WAXMS A capillary column (60 m × 0.25 mm × 0.25 µm film thickness; Thermo Fisher Scientific, Bartlesville, OK, USA). The volatile compounds injected into the inlet were delivered to the column at a splitless mode and helium was used as a carrier gas at a constant flow rate 1 mL/min. The oven temperature program was as follows: initial temperature of 40 °C was maintained for 5 min, increase 2 °C/min to 210 °C and hold for 10 min. The MS spectra were recorded in the electron impact ionization mode (EI) at an ionization energy 70 eV. The mass spectrometer was performed in full scan mode in the range 30–300 *m/z*. The data obtained were processed using Chromeleon^TM^ Data System (Thermo Fisher Scientific Inc., Bartlesville, OK, USA). Identification of volatile compounds was achieved by comparing the recorded mass spectrum with the data available in Wiley Registry 12th Edition/NIST Spectral Library. The Retention index (RI) was calculated using alkane standards C_8_–C_20_ (Sigma Aldrich, St. Loius, MO, USA) according to the equation described in Song, et al. [25] and compared with previously reported in the literature [39,40] (Supplemental Tables S1 and S2).

### 3.3. Optimization of SPME-Arrow Method for Determination of Free VOCs

Before setting the levels of the studied factors, some preliminary experiments using one-factor-at-the-time methodology were necessary.

#### 3.3.1. Determination of Sample Weight

For determining the appropriate sample weight, the weight of 100 mg, 300 mg and 500 mg were used. To determine the injection mode on a GC/MS instrument, the split and splitless (1:5) mode was performed. The sorption conditions were as follows: the appropriate sample weight was incubated at 60 °C for 10 min and then the CWR/PDMS (120 µm × 20 mm) SPME-Arrow was exposed for 30 min. Desorption was done at 250 °C for 5 min. After desorption Arrow was conditioned at 250 °C for 10 min. The SPME-Arrow sorption conditions were chosen based on the previously reported research on the freeze-dried plant material [22]. All experiments were conducted in triplicate.

#### 3.3.2. Selection of SPME-Arrow Coating

The selection of fibre coatings was carried out by testing five commercially available SPME fibres (Thermo Fisher Scientific Inc., Brookfield, OK, USA), and are as follows: PA (100 µm × 20 mm), PDMS (100 µm × 20 mm), DVB/PDMS (120 µm × 20 mm), CWR/PDMS (120 µm × 20 mm), and DVB/CWR/PDMS (120 µm × 20 mm). The sorption conditions were as follows: 100 mg of sample was incubated at 60 °C for 10 min and then appropriate SPME-Arrow was exposed for 30 min. Desorption was done at 250 °C for 5 min. After desorption Arrow was conditioned at 250 °C for 10 min. All experiments were conducted in triplicate.

### 3.4. Experimental Design and Statistical Analysis

To optimise the SPME-Arrow extraction conditions Box-Behnken experimental design (BBD) and response surface methodology were used. Sample weight and SPME-Arrow coating were constant: 100 mg for sample weight, and DVB/CWR/PDMS. Desorption was done at 250 °C while conditioning was done at 250 °C for 10 min. The variables selected for SPME-Arrow optimization were the extraction temperature, incubation time, exposure time, and desorption time (Table 5). In total 27 experiments were generated by BBD and were executed in randomized order. For establishing the optimum conditions for individual classes of VOCs the analysis of variance (ANOVA), regression and plotting of the response surface plot were conducted. For optimization multicriteria methodology (Derringer function or desirability function) was used. This methodology is used when various responses must be considered at the same time, and it is necessary to find optimal compromises between the total number of considered responses [41]. The analysis of the experimental design and calculation of the predicted data was completed using the Design Expert software (Stat-Ease Inc., Minneapolis, USA).

### 3.5. Acid Hydrolysis

In order to analyze bound volatile organic compounds, the free VOCs had to be removed from the sample. The removal of free VOCs was carried out by heating the intact grape skins powder at 60 °C. To test the time needed for removal of free volatiles, the times 1, 2, 3, and 4 h were selected, along with control (no heating 0 h). The heating was carried out on a magnetic stirrer (RTC basic, IKA, Staufen, Germany). To confirm the removal of free volatiles, the sample was analyzed by SPME-Arrow-GC/MS.

The conditions of acid hydrolysis were based on the work of Perestrelo, et al. [12] with minor adjustments. In short, 100 mg of grape skin powder (free VOCs removed) was placed in 20 mL headspace screw-top vials. Acid hydrolysis was carried out by reconstituting the dry sample in 4 mL of citric acid buffer (pH 2.4). The vials were sealed with PTFE/silicone septum containing caps and placed in a thermostated bath adjusted at 95 °C for 2 h in the nitrogen atmosphere. Immediately after hydrolysis, the samples were cooled down in an ice bath and salted out with 2 g of NaCl.

To optimise the SPME-Arrow extraction conditions Box–Behnken experimental design (BBD) and response surface methodology were used. Sample weight and SPME-Arrow coatings were constant and were 100 mg and DVB/CWR/PDMS. The variables selected for SPME-Arrow optimization were the extraction temperature, incubation time, and exposure time (Table 6). All applied statistical methods were the same as described in the Section 3.4.

### 3.6. Method Validation

Validation of optimized SPME-Arrow method for bound VOCs was performed by means of 20 compounds (Supplemental Table S3) belonging to the following groups: aldehydes (4 compounds), alcohols (6 compounds), C_13_-norisoprenoids (2 compounds), acids (4 compounds), and monoterpenes (4 compounds). Calibration curves were constructed using five different concentrations of standards in citric buffer (pH 2.4). The linearity of analyzed compounds was completed by estimation of the regression curves and expressed by the squared determination coefficient (R^2^). The limit of detection (LOD) was defined, according to IUPAC, as the smallest amount of analyte concentration in the sample that can be reliably distinguished from zero, with the acceptance criteria that signal-to-noise (S/N) ratio is 3. The limit of quantitation (LOQ) is the lowest amount of analyte in the sample, which can be quantitatively determined with suitable precision and accuracy, with the acceptance criteria that the S/N ratio is 10. The intraday precision was determined from 3 successive injections of the mix of standard compounds prepared in citric acid buffer (pH 2.4). The interday precision was determined by 3 injections on 3 different days of the week. The precision was calculated using the relative standard deviation (RSD %).

## 4. Conclusions

The SPME-Arrow method is a novel extraction technology which has been employed in the analysis of VOCs from grape skins. This method has proven to be fast and efficient for the analysis of both free and bound VOCs from grape skins. In this study the evaluation of five commercially available fibre coating materials was done and determined that DVB/CWR/PDMS coating is superior in the extraction efficiency compared to other fibres. A total of 53 free and 84 bound VOCs were analyzed and identified by SPME-Arrow method. Since the SPME-Arrow is very sensitive to experimental conditions, the process was optimized by employing Box-Behnken experimental design. For free VOCs the optimum extraction conditions are: extraction temperature 60 °C, incubation time 20 min, exposure time 49 min, and desorption time 7 min at 250 °C. Optimum extraction process for bound VOCs includes removal of free volatiles by heating of the sample, followed by acid hydrolysis. The optimum SPME-Arrow conditions are: extraction temperature 60 °C, incubation time 20 min, exposure time 60 min, desorption time 7 min at 250 °C. Application of the optimized method provides a powerful tool for establishment of the global volatile profile of grape skins. Furthermore, the method is automatized and can be applied to a large number of samples for expeditious analysis of major classes of volatile organic compounds in grape skins.

## Figures and Tables

**Table 1:** Coefficients of the second order polynomial equation and regression coefficients of response and ANOVA parameters for obtained models for free volatiles.

Terms	Aldehydes		Alcohols		Acids		Ketones		Monoterpenes		Sesquiterpenes	
	Coefficient	*p*-Value	Coefficient	*p*-Value	Coefficient	*p*-Value	Coefficient	*p*-Value	Coefficient	*p*-Value	Coefficient	*p*-Value
Model		<0.0001		<0.0001		<0.0001		0.0332		0.0235		<0.0001
Lack of Fit		0.5139		0.5554		0.4554		0.9861		0.9949		0.8611
Intercept	1.44 × 10^10^		3.71 × 10^10^		5.84 × 10^9^		5.18 × 10^9^		1.09 × 10^9^		5.80 × 10^9^	
A-Temperature	4.62 × 10^9^	<0.0001	1.11 × 10^10^	<0.0001	1.66 × 10^9^	<0.0001	−56,416.67	0.0403	4.10 × 10^8^	0.0001	3.92 × 10^9^	<0.0001
B-Incubation	6.85 × 10^8^	0.0761	1.29 × 10^9^	0.2066	2.18 × 10^8^	0.2066	10,134.22	0.9384	29810.39	0.7139	7.56 × 10^8^	0.0040
C-Exposure	1.07 × 10^9^	0.0079	3.05 × 10^9^	0.0063	8.62 × 10^8^	0.0063	2.07 × 10^8^	0.0157	1.64 × 10^8^	0.0412	1.05 × 10^9^	0.0002
D-Desorption	5.68 × 10^8^	0.1047	1.49 × 10^9^	0.1200	2.33 × 10^8^	0.1200	−1.56 × 10^8^	0.2099	61,333.33	0.4160	2.56 × 10^8^	0.2151
AB	−1.75 × 10^8^	0.7600	−1.93 × 10^9^	0.2342	−1.16 × 10^8^	0.2342	−2.02 × 10^8^	0.3419	−25,250.00	0.8446	47807.50	0.8900
AC	−2.90 × 10^8^	0.0140	−3.24 × 10^8^	0.0370	4500.00	0.0570	−4.27 × 10^8^	0.0582	−24000.00	0.0422	3.15 × 10^8^	0.0397
AD	1.73 × 10^8^	0.7633	−60000.00	0.9696	−1.15 × 10^8^	0.9696	−1.95 × 10^8^	0.3583	85,750.00	0.5094	2.35 × 10^8^	0.5000
BC	25880.06	0.9712	2.59 × 10^9^	0.2043	1.15	0.2043	−21,152.65	0.9354	−5681.18	0.9719	−4.61 × 10^8^	0.2985
BD	3.05 × 10^8^	0.5960	−1.51 × 10^9^	0.3447	4.26 × 10^8^	0.3447	1.98 × 10^8^	0.3518	−1.54 × 10^8^	0.2469	−7.86 × 10^8^	0.0387
CD	15000.00	0.9791	−1.43 × 10^9^	0.3709	−1.20 × 10^8^	0.3709	100,000.00	0.6326	−76,750.00	0.5541	−3.19 × 10^8^	0.3640
A²	5.78 × 10^8^	0.2481	−22,087.99	0.9868	4.44 × 10^8^	0.9868	−4.41 × 10^8^	0.0257	−60,420.60	0.5834	6.93 × 10^8^	0.0331
B²	1.54 × 10^8^	0.7642	−4.91 × 10^8^	0.7279	3.67 × 10^8^	0.7279	−1149.79	0.9951	−1.10 × 10^8^	0.3493	2.86 × 10^8^	0.3643
C²	−2.17 × 10^8^	0.6746	−1.33 × 10^9^	0.3559	−2.69 × 10^8^	0.3559	−4.24 × 10^8^	0.0391	−2.18 × 10^8^	0.0782	−1.23 × 10^8^	0.6934
D²	−4.82 × 10^8^	0.3314	−1.92 × 10^9^	0.1683	1.28 × 10^8^	0.1683	−89,476.12	0.6151	−2.07 × 10^8^	0.0776	−3.63 × 10^8^	0.2310
*R^2^*	0.9501		0.9379		0.9408		0.9640		0.9919		0.9752	
Adapted *R*^2^	0.8918		0.8655		0.8655		0.8719		0.8490		0.9463	
Precision	124.7404		123.606		123.606		55.799		62.073		196.936	

**Table 2:** Optimal SPME-Arrow extraction conditions, predicted and experimentally obtained values for individual groups of free volatile compounds.

Group	Temperature (°C)	Incubation Time (min)	Exposure Time (min)	Desorption Time (min)	Predicted Value (Peak Area × 10^6^)	Obtained Value (Peak Area × 10^6^, Mean ± SD)
Aldehydes	60	20	49	7	293.00	298.00 ± 2.50
Alcohols					48.00	47.50 ± 0.95
Ketones					4.80	4.76 ± 0.05
Acids					8.34	8.39 ± 0.09
Monoterpenes					1.32	1.37 ± 0.02
Sesquiterpenes					10.30	11.00 ± 0.31

**Table 3:** Coefficients of the second order polynomial equation and regression coefficients of response and ANOVA parameters for obtained models for bound volatiles.

Terms	Alcohols		Acids		Carbonyls		Norisoprenoids		Monoterpenes	
	Coefficient	*p*-Value	Coefficient	*p*-Value	Coefficient	*p*-Value	Coefficient	*p*-Value	Coefficient	*p*-Value
Model		0.0064		<0.0001		0.0364		0.0008		0.0012
Lack of Fit		0.6491		0.6201		0.7224		0.2524		0.6034
Intercept	2.29 × 10^11^		2.30 × 10^10^		3.10 × 10^10^		4.28 × 10^8^		8.37 × 10^9^	
A-Temperature	−2.92 × 10^10^	0.0027	1.40 × 10^10^	<0.0001	3.83 × 10^9^	0.0436	6.14 × 10^8^	<0.0001	4.02 × 10^9^	<0.0001
B-Incubation	−9.16 × 10^9^	0.1450	−4.94 × 10^8^	0.4751	3.89 × 10^9^	0.0415	12,522.50	0.7835	−2.35 × 10^8^	0.4811
C-Exposure	−1.60 × 10^10^	0.0296	8.24 × 10^9^	<0.0001	6.86 × 10^9^	0.0048	2.73 × 10^8^	0.0015	2.15 × 10^9^	0.0009
AB	2.03 × 10^9^	0.7981	−1.13 × 10^9^	0.2686	−4.94 × 10^8^	0.8162	−24,545.00	0.7043	−7.39 × 10^8^	0.1516
AC	−6.91 × 10^9^	0.0397	6.64 × 10^9^	0.0007	−1.98 × 10^9^	0.1424	1.99 × 10^8^	0.0226	1.32 × 10^9^	0.0293
BC	5.68 × 10^9^	0.4833	−3.58 × 10^8^	0.7089	1.19 × 10^9^	0.5796	−20,000.00	0.7566	−32425.00	0.9437
A²	−1.62 × 10^10^	0.0936	2.25 × 10^9^	0.0622	4.40 × 10^8^	0.8424	2.00 × 10^8^	0.0255	4.44 × 10^9^	0.3735
B²	1.02 × 10^10^	0.2482	−1.80 × 10^9^	0.1141	4.81 × 10^9^	0.0706	1.24 × 10^8^	0.1090	1.46 × 10^9^	0.7614
C²	9.63 × 10^8^	0.9067	1.57 × 10^9^	0.1557	−4.57 × 10^8^	0.8364	−7315.00	0.9129	−1.93 × 10^9^	0.6885
*R^2^*	0.9095		0.9931		0.8991		0.9816		0.9788	
Adapted *R^2^*	0.8467		0.9806		0.9176		0.9484		0.9407	
Precision	89.161		317.821		87.989		177.890		172.746	

**Table 4:** Optimal SPME-Arrow extraction conditions, predicted and experimentally obtained values for individual groups of bound volatile compounds.

Group	Temperature (°C)	Incubation Time (min)	Exposure Time (min)	Predicted Value (Peak Area × 10^6^)	Obtained Value (Peak Area × 10^6^, Mean ± SD)
Alcohols	60	20	60	202.00	209.00 ± 9.14
Acids				49.30	49.90 ± 1.35
Carbonyls				40.20	39.80 ± 0.49
Norisoprenoids				1.53	1.47 ± 0.15
Monoterpenes				15.50	16.20 ± 0.89

**Table 5:** Independent factors and their levels used in the BDD for optimization of SPME-Arrow extraction for determination of free volatile compounds.

Factors	Factor Levels		
Coded levels	−1	0	1
A: Extraction temperature (°C)	40	50	60
B: Incubation time (min)	10	20	30
C: Exposure time (min)	30	45	60
D: Desorption time (min)	5	7.5	10

**Table 6:** Independent factors and their levels used in the BBD for optimization of SPME Arrow extraction for determination of bound volatile compounds.

Factors	Factor Levels		
Coded levels	−1	0	1
A: Extraction temperature (°C)	40	50	60
B: Incubation time (min)	10	20	30
C: Exposure time (min)	30	45	60

## Data Availability

Data sharing not applicable.

## Supplementary Materials

The following are available online. Table S1: Parameters of the identification and physio-chemical properties for free volatile compounds. Table S2: Parameters of the identification and physio-chemical properties for bound volatile compounds. Table S3: Validation parameters of the optimized SPME-Arrow GC-MS method.

## Sample Availability

Not available.
